# Acknowledgment to Reviewers of *Biomimetics* in 2021

**DOI:** 10.3390/biomimetics7010022

**Published:** 2022-01-26

**Authors:** Biomimetics Editorial Office

**Affiliations:** MDPI AG, St. Alban-Anlage 66, 4052 Basel, Switzerland

Rigorous peer-reviews are the basis of high-quality academic publishing. Thanks to the great efforts of our reviewers, *Biomimetics* was able to maintain its standards for the high quality of its published papers. Thanks to the contribution of our reviewers, in 2021, the median time to first decision was 18 days and the median time to publication was 44 days. The editors would like to extend their gratitude and recognition to the following reviewers for their precious time and dedication, regardless of whether the papers they reviewed were finally published:
Adam JunkaJu-young ShinAdrian KampaKatsuhiko ArigaAlbert KrastanovKonstantinos TsiakasAlberto Rodríguez-ArchillaKonstantinos TsongasAlessandro GiorgettiKristina WanieckAlexandrina MunteanKung-Ming ChungAli GhazvinianLana ZoricAlvaro GutierrezLang ZhouAmit NautiyalLeire Ruiz-RubioAndrea RuffiniLiss C. WernerAndrzej PanasLiviu CiupituAnna DettlaffLlopis RodrigoAntonio ApicellaLoris RovedaAntonio BarrientosLouis Jun Ye OngAntonio Carlos De Oliveira JúniorLuciana GiardinoAnuj KumarLuiz Marcos Garcia GonçalvesArtur GonçalvesMagda JanalikovaAustin M. GarnerMahboob AlamAvneesh KumarMarc DesmulliezBárbara FerreiraMarc WeissburgBeth MallardMarcin WysokowskiBogdan GhermanMarco GovoniBrook KennedyMarco Picchi ScardaoniCarlo TorreMaria A. BonifacioCarlos EscobedoMaria Addolorata BonifacioCedric LiauzunMaria HarjaChelladurai KaruppiahMaría Isabel Rodríguez LópezChiachung ChenMaria KalivaChristian WirajaMarie DavidováChristopher Stewart RoseMarkos PetousisDaniela Cruz-MoreiraMarloes PeetersDavid C. JensenMatteo CastronovoDiego Daniel ManavellaMatteo VandoniDoina PislaMatthew McHenryEdward BormashenkoMaycon SedrezEkaterina BaranovaMiguel AmadoEleonora NistorMikhail KotovEoghan CunnaneMikhail SevostyanovFabian WesthauserMin Wook LeeFatih TezokMircea IvanescuFelicia CarotenutoMircea TiereanFiona LouisMiruna StanFlorin OanceaMohammad El KhatibFrancesco FabbrocinoMohammed Salah Al-RadhiFrancisco Alburquerque-SendínMuhammad Azmi Abdul-HamidFrancisco Manuel Braz FernandesNicholas HengGajanan GhodakeNick RoweGeorges RommeNick Taylor BuckGiulio FuscoNúria TorrasGuy BurroughesOriol PonsGyanendra P. JoshiPalak PatelHocine BougdahPaulina KazimierczakHorst BleckmannPaulo BritoHossein OmranyPawel NakielskiHugues Allard-ChamardPeter NiewiarowskiIlaria MiletiPiotr LulińskiIoannis GiannopoulosPiotr WychowańskiIsabella FiorelloRamdayal YadavIván Hernández-PérezRedha TaiarIvan S. RaginovRenata WłodarczykIvan VirgalaRenu Geetha BaiJaap Den ToonderRichard GravesJacek IzydorczykRoberta ToroJanos KanczlerRodica BorciaJean RabaultRoman RakitovJeannette YenRoss N. GillandersJian-Zhang ChenRuxandra BotezJiaquan YuRyo KohsakaJiusheng LiSalvatore GiuffridaJoão ReisSanju GuptaJohn AggasSébastien B.G. BlanquerJose Luis ChiaraSeyyed Mojtaba MousaviJuan Carlos Pomares TorresShintaro SukegawaJuan Esquivel-RodriguezSina AskarinejadJuan Luis Crespo MariñoSudong KimJulian LienhardTanmoy PramanikJuliane MünchVassilis KostopoulosJürgen Weber


